# A Case of a Thoracic Arteriovenous Fistula Between an Intercostal Artery and a Pulmonary Vein

**DOI:** 10.7759/cureus.27682

**Published:** 2022-08-04

**Authors:** Ian K Motie, Bobby Malik, Jenin Jose, Elora Friar, Joel Baker

**Affiliations:** 1 Internal Medicine, Sarasota Memorial Hospital, Sarasota, USA; 2 Clinical Sciences, Florida State University College of Medicine, Sarasota, USA; 3 Hospital Medicine, Sarasota Memorial Hospital, Sarasota, USA

**Keywords:** embolization, esophageal cancer, hemoptysis, arteriovenous fistula, thoracic fistula

## Abstract

Arteriovenous (AV) fistulas are irregular connections between arteries and veins, and thoracic AV fistulas are rarely identified in clinical practice. We report a case of a 56-year-old female with a history of esophageal adenocarcinoma treated with radiation and resection who presented to the hospital due to hemoptysis. She underwent bronchoscopy revealing a tracheobronchial fistula, and esophagogastroduodenoscopy revealing active arterial bleed. Subsequent angiography uncovered an AV fistula between her right T8 intercostal artery and pulmonary vein with a pseudoaneurysm. She underwent coiling of her T8 and T9 intercostal arteries with resolution of her symptoms.

## Introduction

Arteriovenous (AV) fistulas are irregular connections between arteries and veins. AV fistulas bypass their natural connections in capillaries and allow for a direct connection between arteries and veins, which in general are commonly seen in clinical practice, and in appropriate clinical scenarios can act as a vascular access point for hemodialysis. Among subsets of AV fistulas, thoracic AV fistulas are an exceedingly rare finding with limited reports of their presentation outside of a small subset of cases, such as AV fistulas of the thoracic spine, spinal dural AV fistulas, or congenital AV fistulas between an intercostal artery and a brachiocephalic vein [1-3]. Most AV fistulas are discovered incidentally, as they often lack any clinical signs or symptoms to prompt investigation. In this case, we report on a patient with a history of esophageal carcinoma treated with neoadjuvant radiation and resection who presented with hemoptysis. She underwent esophagogastroduodenoscopy (EGD) and was found to have an active arterial bleed. After the culprit vessel was ligated, she underwent an arteriogram that revealed an abnormal communication between her right T8 intercostal artery and right pulmonary vein. The identified fistula was also found to have a pseudoaneurysm. Based on the location of the pseudoaneurysm, it was believed that her pseudoaneurysm began to erode into her esophagus ultimately leading to her presentation with hemoptysis. The patient underwent coiling of her T8 and T9 arteries that resolved her symptoms.

## Case presentation

A 56-year-old female patient presented to the hospital with acute onset hemoptysis. She described episodes where she coughed up blood-tinged sputum and sputum containing coffee ground-appearing substances earlier that day. She denied pain associated with these episodes and never had this symptom prior to presentation. Her past medical history was significant for esophageal adenocarcinoma treated with neoadjuvant radiation and resection. She underwent esophago-gastric anastomosis, complicated with wound dehiscence leading to prolonged post-operative course. Her residual dysphagia led to esophageal stent placement with the most recent stent being removed one week prior to presentation.

**Figure 1:**
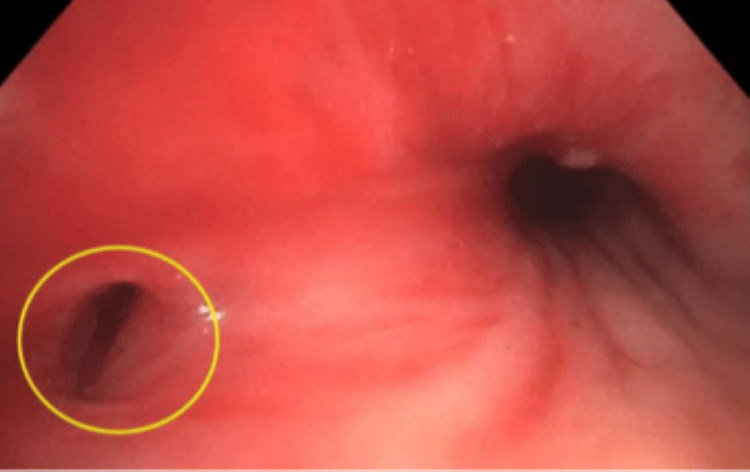
Bronchoscopy depicting the broncho-esophageal fistula in the medial wall of the right mainstem bronchus, circled in yellow

**Figure 2:**
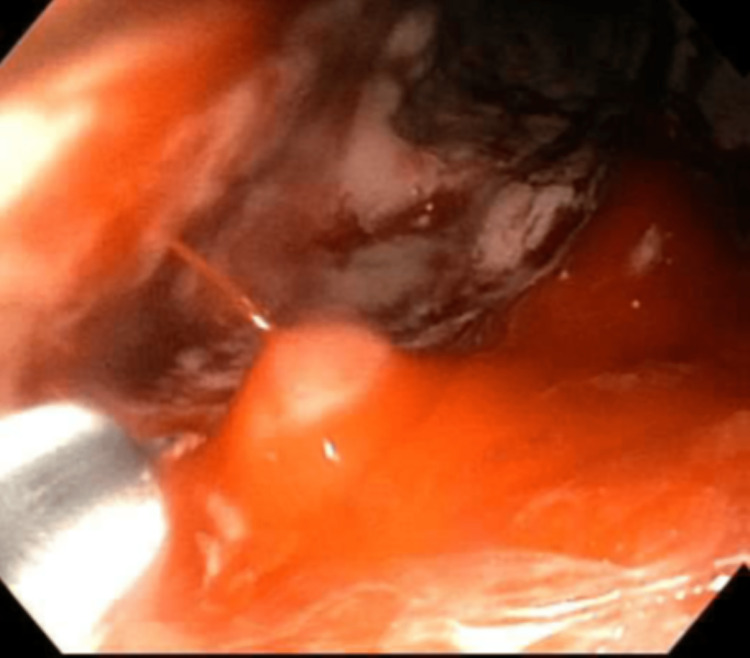
Esophagogastroduodenoscopy depicting active arterial bleeding at the site of the esophago-gastric anastomosis prior to embolization

On presentation, her vital signs were within normal limits. Her physical examination was notable for decreased breath sounds at the right lung base without wheezes, rales, or rhonchi. Laboratory findings revealed hemoglobin at 12.0 g/dL with the remainder of her complete blood count and metabolic profile unremarkable. CT of the thorax demonstrated multifocal nodularities in the right lung. On the first night of her hospitalization, the patient had large volume hematemesis of approximately 6 ounces with a subsequent hemoglobin reading of 9.4 g/dL. She was urgently transfused two units of packed red blood cells and taken for bronchoscopy. This procedure evinced edematous and erythematous bronchial mucosa of the right lower lobe and bronchus intermedius and an abnormal orifice on the medial wall of the right mainstem bronchus consistent with a broncho-esophageal fistula (Figure 1). There was no notable purulence sputum identified during this procedure. She subsequently underwent EGD that showed intact esophago-gastric anastomosis with localized inflammation and an exposed vessel with active arterial spurting after contact (Figure 2). Hemostasis was achieved with epinephrine injection and three hemostatic clips were placed. The patient was then intubated for airway protection and transferred to the intensive care unit.

**Figure 3:**
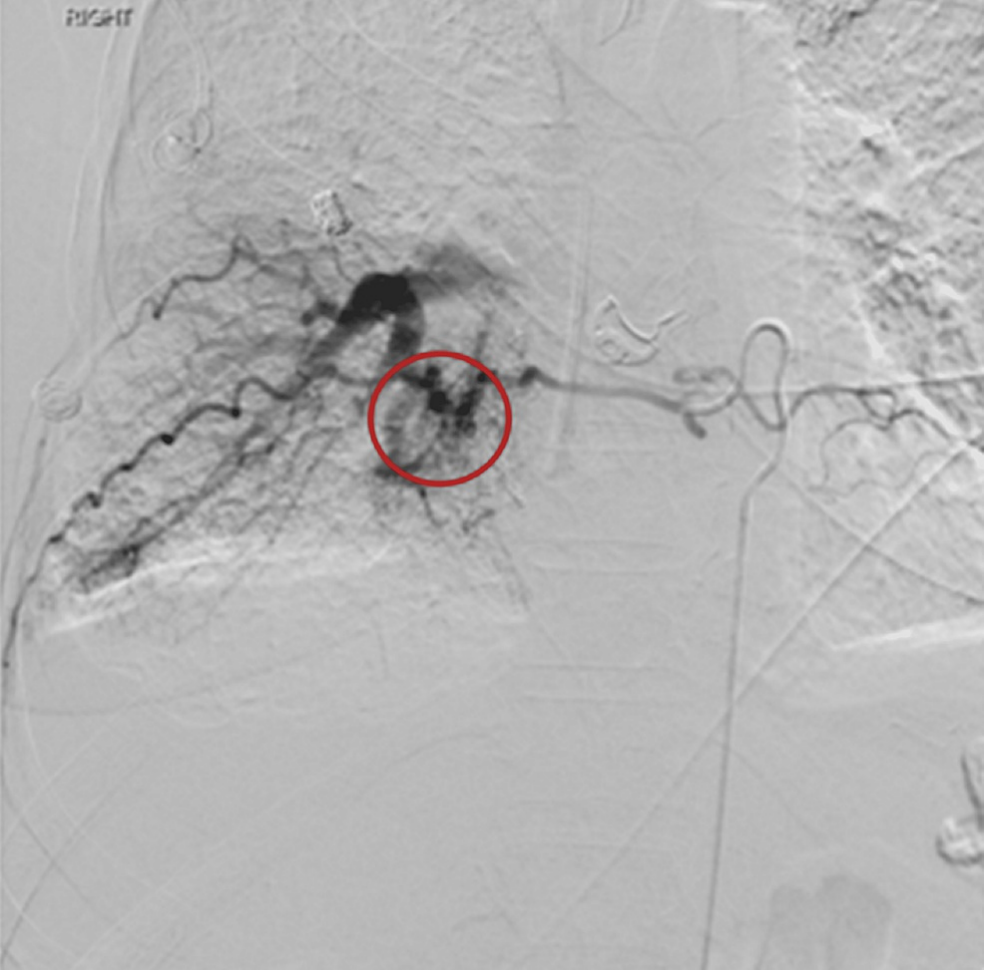
Angiography via catheterization of the right T8 intercostal artery showing communication with the right pulmonary vein, circled in red

**Figure 4:**
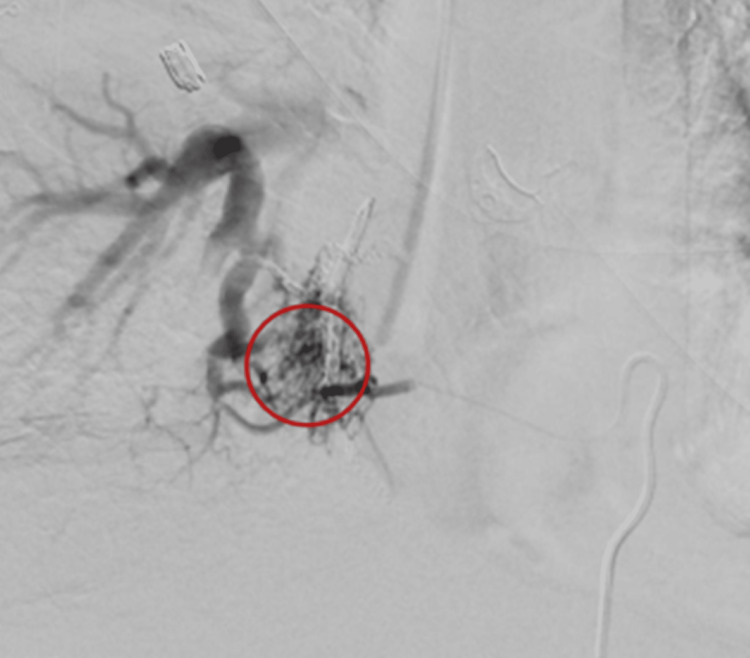
Angiography via catheterization of the right T9 intercostal artery showing communication with the right pulmonary vein, circled in red

In order to determine the location of the bleed, the patient underwent diagnostic angiography with interventional radiology. The proximal descending thoracic aorta was initially accessed that showed immediate pulmonary venous return suggestive of an AV fistula from the right T8 and T9 intercostal arteries or right pulmonary veins. The right T8 intercostal artery was catheterized with studies confirming the presence of an AV fistula between the right T8 intercostal artery to right pulmonary vein (Figure 3). The right T9 intercostal artery was also accessed that further corroborated these findings (Figure 4).

Both arteries underwent coil embolization. Upon further review of the imaging, the presence of an eroding pseudoaneurysm at the site of the AV fistula was favored as the etiology of the patient's hematemesis. Based on the location of the pseudoaneurysm and the patient's altered anatomy following her esophagectomy, it was theorized that her pseudoaneurysm began to erode into her esophago-gastric anastomosis that ultimately led to the patient’s hemoptysis. The patient remained hemodynamically stable after her embolization and had no recurrence of hemoptysis or hematemesis. She was eventually transferred to the medical floor and subsequently discharged with close outpatient follow-up with pulmonology, gastroenterology, and hematology-oncology.

## Discussion

Thoracic AV fistulas are exceedingly rare and are equally difficult to diagnose as the majority of cases are either mild or completely asymptomatic [1-5]. Reviewing the literature, the majority of identified thoracic AV fistulas are most commonly associated with spinal cord vasculature or present as leptomeningeal AV fistulas [1,2]. Based on the location of these fistulas, they often manifest with neurologic symptoms [1,2]. Looking specifically at intercostal arterial fistulas, several reports have identified fistulas as an asymptomatic congenital finding often incidentally found in children [3,4]. One case report identified an arterio-arterial fistula between an intercostal artery and a pulmonary artery [5]. Furthermore, one case reported an almost similar finding with an intercostal artery aneurysm caused by an AV fistula [6].

Specifically looking at the presence of a fistula between an intercostal artery and a pulmonary vein, these findings have been reported in one case report where the patient also presented with hemoptysis. The patient was treated initially with electrocautery and ligation of the culprit vessels, the right T4 and T5 intercostal arteries. However, this intervention did not resolve the patient's symptoms as there was a recurrence of hemoptysis. The treating clinicians opted for treatment with right upper and middle lobectomy, which ultimately resolved the patient's symptoms [7].

In general, fistulas often manifest after localized inflammation and/or trauma between two communicating structures. In our patient, her thoracic AV fistula most likely manifested after her prolonged treatment course for esophageal adenocarcinoma. She endured surgical resection with the formation of a new esophago-gastric anastomosis and unfortunately experienced a complicated post-operative course with wound dehiscence requiring revisions, all of which likely laid the foundation for an AV fistula to form. She likely remained asymptomatic from her AV fistula, but as a pseudoaneurysm formed and expanded near her esophago-gastric anastomosis, its erosion had life-threatening manifestations with hemoptysis and hematemesis.

Comparing both cases, our patient was treated with relatively conservative measures and fortunately did not require extensively invasive interventions such as a lobectomy. While individual patient characteristics likely play a role in the difference between outcomes, our patient never underwent electrocautery or ligation of her intercostal arteries and was ultimately treated with coil embolization. While there is little definitive data comparing outcomes between coil embolization and ligation in this specific clinical scenario, coil embolization in general is a less invasive intervention compared to electrocautery and ligation. In summation, treatment of AV fistulas with severe manifestations such as our patient's could feasibly be treated conservatively with the localization of the culprit fistula and subsequent coil embolization if the patient is hemodynamically stable.

## Conclusions

The vast majority of thoracic AV fistulas usually do not have corresponding clinical manifestations; this further leads to their identification being rare. Given the rare nature of thoracic AV fistulas and the broad spectrum of presentations they are associated with, they often fall lower on the differential diagnosis when determining etiologies for certain pathologies. However, in patients with a history of intrathoracic surgeries or trauma, the presence of a thoracic AV fistula should be actively considered as an etiology.

The approach to identifying the culprit vessels should be stepwise with the first step investigating common reasons for a specific presentation (i.e., EGD for hematemesis) while ensuring the stability of the patient. If an AV fistula is not ruled out with the initial workup, assessing the patient's vascular anatomy with an arteriogram appears to be the most effective means of both identifying and treating the fistula with coil embolization. Patients should then be monitored closely after undergoing coil embolization to ensure there is no recurrence of symptoms. As more cases of thoracic AV fistulas are identified, specifically those with presentations of hemoptysis or hematemesis, there may hopefully be more guidance in a formalized approach to treatment.
